# Innovations Driving the Future of Maxillofacial Prosthetics, Part I: The Technological Leap in Maxillofacial Rehabilitation

**DOI:** 10.1055/s-0045-1809180

**Published:** 2025-05-27

**Authors:** Mostafa I. Fayad, Mohamed Ashour Ahmed, Marwa Kothayer, Mona M. Aboelnagga, Emad El Said Fahim Essa, Osama Abu-Hammad, Rania Moussa

**Affiliations:** 1 Department of Substitutive Dental Sciences, College of Dentistry, Taibah University, Al-Madinah Al-Munawarah, Saudi Arabia; 2 Department of Removable Prosthodontics, Faculty of Dental Medicine, Al-Azhar University (Cairo, Boys), Cairo, Egypt; 3 Department of Prosthodontics, College of Dentistry, Taif University, Taif, Saudi Arabia; 4 Department of Oral and Maxillofacial Prosthodontics, Faculty of Dentistry, Ain Shams University, Cairo, Egypt; 5 Oral and Maxillofacial Surgery Diagnostic Science Department, College of Dentistry, Taibah University, Al-Madinah Al-Munawarah, Saudi Arabia; 6 Department of Oral and Maxillofacial Surgery, College of Dentistry, Tanta University, Tanta, Egypt; 7 Department of Prosthodontics, Faculty of Dentistry, University of Jordan, Amman, Jordan

**Keywords:** maxillofacial prosthesis, digital technology, artificial intelligence, augmented reality, 3D printing, virtual reality

## Abstract

Maxillofacial prosthetics is a specialized field focused on replacing part or all of the facial and cranial structures. In recent years, digital technology has gained popularity over traditional methods for fabricating maxillofacial prostheses. This study aimed to provide a comprehensive review of recent innovations in maxillofacial prosthetic rehabilitation, with an emphasis on emerging technologies and their impact on patient outcomes, treatment efficiency, and overall quality of life.

A comprehensive literature search was conducted across the Web of Science, PubMed, MEDLINE, and CENTRAL databases for studies published in English within the last decade. The keywords utilized included “Maxillofacial Prosthesis Implantation,” “Maxillofacial Prosthesis,” and “rehabilitation,” as well as “Virtual Rehabilitation,” “Artificial intelligence,” and “digital technology.” The initial search yielded 178 articles. After resolving 51 duplicates, 127 articles were screened based on titles and abstracts. Following full-text assessment, 81 articles met the inclusion criteria and were included in the review.

The results indicated that advancements in digital technologies, digital imaging, data acquisition, and the integration of digital workflows have significantly transformed the rehabilitation of maxillofacial prosthetics. These innovations enabled customization and personalization, provided an improved fit, enhanced precision, reduced number of visits and chair time, and facilitated seamless rehabilitation of complex maxillofacial defects. Four-dimensional printing involves materials that can change shape or properties over time, enabled printed objects to adapt dynamically to external stimuli, enhancing both comfort and functionality of prosthetics. Further advancements, such as five-dimensional and six-dimensional printing, improved the sensory capabilities of prostheses. Virtual and augmented reality enhance real-world experiences by overlaying digital data, improving accuracy and fit, enabling virtual surgical planning, and developing patient-specific implants. Artificial intelligence (AI) assists automated decision-making and supports the design of AI-driven prostheses. AI algorithms have shown the potential to automate digital planning, replicate intricate anatomical features, and attain high diagnostic accuracy in maxillofacial prosthodontic scenarios.

## Introduction

Maxillofacial prosthetics is a specialized prosthodontics field that replaces part or all of the stomatognathic and craniofacial structures. 1 It intends to restore form and function, enhance aesthetics, and improve the overall quality of life. Patients experience maxillofacial deformities because of trauma, cancer, or congenital disabilities. 2

Maxillofacial prostheses have a long history, dating back to ancient civilizations, with documented records of their use. Mummified Egyptians were found buried with enamel-covered silver eyes with bronze lids as well as nasal and auricular structures. Evidence from ancient Greek, Chinese, and Indian civilizations was also documented. In the 16th century, French surgeons were pioneers in the use of artificial eyes, ears, noses, and obturators, laying the groundwork for the field. 3

Over the years, materials such as paper-mâché, leather, ivory, gold, and silver were used to create these prostheses, while later glass and wood became common materials. In the 17th century, Pierre Fauchaud described a palatal obturator with foldable wings for insertion. In the 19th century, William Morton used gold obturators and porcelain artificial noses. By the end of the 19th century, vulcanite had been introduced, along with other materials such as celluloid, gelatin, glycerin-based materials, and resins. 2

The 20th century witnessed maxillofacial prostheses primarily focusing on restoring cleft palate, apart from during the First and Second World Wars, when a high demand for facial prostheses was reported, leading to the development of specialized maxillofacial units in the United Kingdom. 4

The development of maxillofacial prosthetics continued to evolve and involve diverse prosthetic devices functioning intra- and extraorally, including obturators, orbital, nasal, auricular, and facial prostheses. Because of the associated aesthetic and psychological issues, such prostheses commonly require high-quality precision, efficiency, and personalization, thus improving patient outcomes and boosting quality of life. 5

Over the years, maxillofacial prosthetic rehabilitation has undergone significant transformation due to advancements in digital technologies, materials, and communication. Modern prosthodontists now have access to advanced tools and techniques. Currently, maxillofacial prostheses are fabricated using acrylic resins, silicones, and advanced biomimetic materials. They are retained and supported by teeth, osseointegrated implants, remaining skin with or without adhesive, and body cavities. 2 6

Maxillofacial prosthetics were typically fabricated using conventional impressions and hand-sculpted wax patterns, followed by flasking and packing silicone or polymethyl methacrylate, and a trial-and-error fitting process. In contrast, intraoral and extraoral scanners enable precise three-dimensional (3D) imaging of defect areas. The cone-beam computed tomography (CBCT) machine is a well-established adjunctive diagnostic, virtual simulation, and treatment planning tool, integrating CBCT scans with surface scans enables more accurate surgical and prosthetic planning. 2 7

Digital approaches widened the range of materials used, including medical-grade silicones printed with specialized 3D printers, resins for molds or prototypes, and titanium or polyetheretherketone frameworks printed via selective laser melting (SLM) or other metal 3D printing (3DP) techniques. These digital materials and technologies typically offer improved biocompatibility, color matching, and mechanical properties compared to traditional options, leading to a more precise fit. 8

However, these digital advancements also come with limitations. The initial cost of equipment and training can be high, and access to these technologies may be limited in certain regions. Additionally, some digital materials may not be as aesthetically pleasing or durable as conventional silicones, and there can be challenges in color-matching complex facial features with specific digital systems. Finally, there is a need for technician expertise in both digital and artistic domains. 9

This review aimed to summarize the latest innovations in maxillofacial prosthetics, focusing on emerging digital technologies. It provides an overview of recent rehabilitation techniques in this field, presenting their applications, benefits, barriers, and associated incentives. Part II will discuss the pioneering innovations in materials, biomaterials, and regenerative therapies.

## Methods

To conduct this scoping review, a thorough literature search was conducted in electronic databases without constraints on regions or publication types to identify relevant studies on maxillofacial prosthetics. The primary sources were the electronic databases of Web of Science, PubMed, MEDLINE, and ProQuest CENTRAL. The search applied the following Medical Subject Headings (MeSH) terms: (“Maxillofacial Prosthesis Implantation” OR “Maxillofacial Prosthesis” OR ” Maxillofacial rehabilitation”) AND (“Virtual rehabilitation” OR “Augmented reality” OR “Artificial intelligence,” OR “Biotechnology” OR “Digital technology”). Search terms were customized for each database to account for variations in indexing practices and search syntax. In addition to systematic database searches, a manual search of reference lists and relevant journals was conducted to identify further potentially eligible studies.

A modified PICO framework was used to define the research question. PICO stands for: Patient/Problem (P): The population or condition of interest; Intervention (I): The treatment, technology, or approach being investigated; Comparison (C): The alternative intervention or standard practice; Outcome (O): The desired result or effect of the intervention. Alternatively, the review question was framed as follows: For patients requiring maxillofacial prostheses (P), what are the latest digital technologies and materials used in their fabrication (I), and what are the reported benefits and limitations (O).

Based on the above, this review's inclusion criteria included randomized clinical, pilot, cross-sectional, and retrospective studies published in peer-reviewed journals in the last 10 years, from January 2015 up to October 2024. The review identified studies exploring the latest technology in maxillofacial rehabilitation. The key innovations encompass digital design and manufacturing, as well as virtual (VR) and augmented reality (AR) and data acquisition technologies. Additionally, the review covered the emerging role of artificial intelligence (AI) and machine learning algorithms. Exclusion criteria included non-English studies published over 10 years ago and were not published between January 2015 and October 2024.

Two authors (M.A.A. and M.K.) conducted database searches. Authors (M.M.A. and E.F.E.) evaluated the titles, abstracts, and relevant articles. R.M. and M.F. screened full texts, with eligibility assessment conducted independently. Any disagreement among reviewers was resolved through discussion, and a third reviewer (O.A.H.) was consulted to resolve any remaining debates.

## Results

The initial search yielded 178 articles. After resolving 51 duplicates, 127 articles were screened based on titles and abstracts. Following full-text assessment, 80 articles met the inclusion criteria and were included in the review.

**Fig. 1:**
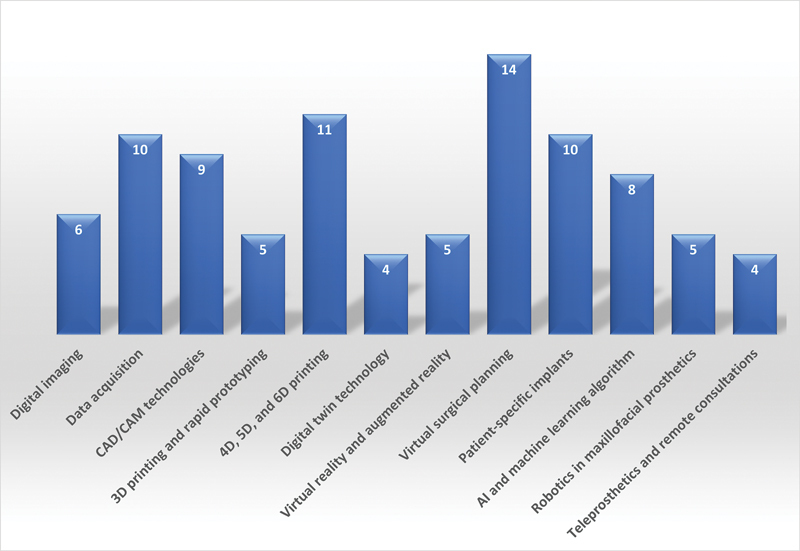
Bar chart comparing the number of selected research papers studying digital technological innovations in maxillofacial prosthetics.

The number of selected articles and the type of digital technology and innovations used in maxillofacial prosthetic rehabilitation are presented in Fig. 1 .

## Discussion

Maxillofacial prosthetics has witnessed significant technological advancements in recent years, particularly integrating digital tools and AI into clinical and laboratory workflows. This research reviewed the key innovations in maxillofacial prosthetic technology, which have transformed the design, fabrication, and delivery of prostheses. The following subsections explore developments in digital imaging and modeling, computer-aided design and manufacturing (CAD/CAM) applications, 3DP technologies, and the role of AI-assisted planning.

### Digital Imaging

Recent advances in medical imaging scanning technologies, including CBCT, digital CT, and magnetic resonance imaging (MRI), produce files in the Digital Imaging and Communication in Medicine format. These files must be converted and saved as STL (Standard Tessellation Language) files and then used to construct adaptable 3D models of the patient's unique anatomical features for maxillofacial rehabilitation. 10 The STL model of the prosthesis can be modified using CAD software. Then, it can be printed using 3DP techniques such as selective laser sintering (SLS) and fused deposition modeling (FDM). 11 These technologies are particularly effective for complex cases, such as auricular and orbital defects. 12

Intra- and extraoral scanning enhanced prosthetic design and fit accuracy by capturing the defect's topography and superficial structures of the teeth and soft tissues and transferring them directly into 3D STL files. 13 These imaging modalities allowed for precise 3D representations of complex anatomical structures, which have been utilized to determine the optimal location for implant placement, particularly in patients with uncertain bone quality or quantity. Thus, they facilitate the planning and execution of prosthetic devices. 14

Wang et al 11 employed a digital approach to rehabilitate facial deformities resulting from maxillofacial trauma. The process involved gathering data through CBCT imaging and 3D facial scans. ProPlan CMF software generated a virtual model representing the patient's craniofacial hard tissues, realistic facial soft tissues, and existing dentition. This digital workflow successfully predicted aesthetic outcomes for patients with maxillofacial trauma, enhanced communication with patients, decreased clinical time, and informed the collective design of implant placement and prosthetic restoration fabrication.

Vosselman et al 15 presented a digitized surgical planning workflow for single-stage maxillectomy procedures and zygomatic implant placement, utilizing 3D-printed cutting and drilling guides. This approach enabled the immediate insertion of zygomatic implants and the simultaneous fabrication of an implant-supported obturator prosthesis, demonstrating high positional precision and prosthetic fit.

### Data Acquisition

Data acquisition is the initial and most crucial step, as it determines the outcome of the prostheses. Various surface scanners, such as intraoral, structured light, laser, and facial scanners, are reliable methods for defect data acquisition. 16

Corsalini et al 17 conducted a randomized controlled trial and demonstrated that digital impressions in implant prosthetic rehabilitation showed significantly better interproximal and occlusal contact, less impression time, and enhanced patient satisfaction. In contrast, no significant differences were reported for the abutment-implant fit.

Digital scanners capture highly detailed 3D representations of the patient's anatomy, reducing the discomfort and distortion risks associated with conventional impression-taking. They also capture precise topographical data without direct tissue contact, thus eliminating the risk of interoperator variability. The acquired digital data can then be seamlessly integrated into CAD/CAM software, streamlining prosthetic rehabilitation's design and manufacturing phases through various rapid prototyping (RP) techniques. 16

The clinical outcomes of laser scanners and digital imaging have influenced the preferred digital techniques in various clinical applications. According to Suresh et al, 12 laser scanning systems are extensively utilized in fabricating nasal prostheses, while combining CT scans and digital photography is preferred for auricular prostheses. Additionally, digital photography and stereophotogrammetry are the preferred methods for large facial defects, and the combination of MRI and CT scans appears to be a superior approach for data acquisition for obturators.

Aponte-Wesson et al 18 utilized digital impressions to rehabilitate a patient with bilateral maxillectomy and extensive tissue loss for retention and support. The digital impressions enabled capturing three opposing sinus undercuts to retain, support, and stabilize a hollow one-piece obturator prosthesis.

A facial scanner is a noncontact optical tool that can capture 3D facial models with skin texture and color in an open data format. Face scanners allow facial recognition, emotion capturing, aesthetic planning, and maxillofacial rehabilitation. The accuracy of facial scanners is crucial, and studies have found differences between stated and actual accuracy, especially for patients with midfacial deformities. 19

Stereophotogrammetry, the extraction of 3D features from two-dimensional images of anatomical defects taken from different viewpoints, is a reported alternative to producing 3D surface models of patients' faces. However, the images' resolution is low, and finer skin details are not recorded. 20

The primary beneficial outcomes of digital data acquisition technologies in the treatment planning of prosthetic rehabilitation for maxillofacial defects included reduced fabrication time, as well as improved dimensional accuracy. Aesthetics, dimensional accuracy, and patient satisfaction were highly recognized outcomes in facial prostheses. 12

Baghani et al 21 conducted a study to evaluate the accuracy of digital impression techniques based on trueness and precision in capturing implant-level impressions for maxillofacial prostheses. Two intraoral scanners (TRIOS 3 and CS 3700) and one desktop extraoral scanner (Open Technology) were compared using a reference model simulating an auricular defect. The results revealed statistically significant differences in accuracy among the scanners. In some measurements, the TRIOS 3 scanner showed superior performance compared to the Open Technology and CS 3700 scanners. However, in other measurements, the Open Technology scanner outperformed the intraoral options. The CS 3700 scanner consistently showed higher deviation in several comparisons. Despite variability in scanner performance, the authors concluded that digital impression techniques, both intraoral and extraoral, can serve as viable alternatives to conventional methods for maxillofacial prosthesis fabrication.

Hatamleh et al 22 conducted a cross-sectional study to evaluate the impact of digital technologies on the provision of maxillofacial prosthetic services and to assess patient attitudes and perceptions. The study included 37 patients with facial defects who received prosthetic rehabilitation at an ear, nose, and throat clinic. The patients reported that their prostheses were comfortable, easy to handle, and confidence-enhancing, with most wearing them for over 12 hours daily. So, digital technologies significantly enhance the fabrication process and patient experience, supporting improved satisfaction, functionality, and acceptance of maxillofacial prostheses.

### Computer-Aided Design and Manufacturing

Digital imaging techniques provide detailed anatomical information, which forms the foundation for CAD/CAM processes. These processes utilize software to design and manufacture products. This allows for precise and customized prosthetic solutions. Furthermore, virtual simulations of prosthetic rehabilitation enable clinicians to assess and refine treatment plans before physical fabrication, thereby optimizing outcomes and reducing the need for iterative adjustments. 14

Digitally designed prosthetics significantly reduce production time and improve the final product's quality. This efficiently produces high-quality, patient-specific prostheses, reduces the time required for fabrication, and minimizes human error. 23 24 25

CAD/CAM produces various surgical assistant tools, such as stereolithography (SLA) models, surgical guides, and patient-specific implants (PSIs), which can facilitate the transition from virtual to real. SLA models are CAD/CAM-generated replicas of the craniomaxillofacial bone and planned bone transplants, which serve as templates for preoperative planning, intraoperative checks, patient education, and training. Surgical guides such as repositioning guides, occlusal splints, osteotomy (cutting) guides, and combined predrilling/osteotomy transmit the virtually planned operation to the surgical site in computer-aided surgeries. 26 27

Costa-Palau et al 28 used the digital workflow to restore a large midfacial defect in the right cheek and lip, applying the virtual planning, designing, and manufacturing of the prostheses through 3D silicone printing. The digital prosthesis had acceptable esthetics and fit with enhanced patient satisfaction, especially regarding efficiency, comfort, and delivery time.

Ali et al 29 combined the digital workflow with the conventional to produce the definitive obturator in an anterior maxillectomy patient. Digital scanning was performed for the oral condition and the available interim obturator. Then, CAD/CAM technology was used to fabricate a definitive metal framework obturator. The technique enhanced patient adaptation and ensured more comfortable and reliable clinical procedures.

### 3D Printing and Rapid Prototyping

3DP is an additive manufacturing (AM) process that creates 3D objects by organizing layers of the used materials on top of each other. It encompasses various technologies, including FDM, SLS, and SLA, silicone 3DP, and powder printing/ binder jetting. 16

The field has witnessed remarkable advancements since Charles Hull's pioneering work in SLA-based 3DP in 1986. AM, also known as 3DP, creates physical objects from 3D digital models by depositing or solidifying materials layer by layer. 30

RP is an application used in manufacturing to create a model faster than the normal process. RP is mainly completed using 3DP or AM technology. 31

Reconstructing the facial skeleton, addressing asymmetry, restoring orbital volume, reducing displaced fractures, and enhancing aesthetics and functionality can be complex. The precise transfer of surgical planning from the virtual environment to the actual surgical setting is critical in computer-assisted surgery and is essential for achieving clinically satisfactory accuracy. 32

AM technologies, such as extrusion and photopolymerization, enable the fabrication of customized designs with intricate structural complexity and rapid and cost-effective delivery. 3DP also allows the production of biocompatible materials that can enhance the durability and functionality of prosthetic devices. Common materials utilized in SLA models include polyamide, silicone, and acrylics, with the latter offering the ability to be transparent or dyed to highlight specific structures. 33

### Four-, Five-, and Six-Dimensional Printing

The integration of four-dimensional (4D) printing involves materials that can change shape or properties over time, and this field is also being explored as a future advancement. 34

4D printing is based on adding a fourth dimension to standard 3DP by adding the element of time or self-transformation. The printed object changes shape or function in response to external stimuli such as heat, moisture, light, or magnetic fields. 35 36

Thermoresponsive materials [e.g., poly ( *N* -isopropyl acrylamide), gelatin, collagen, poly(ether urethane)] are frequently employed materials in 4D printing platforms. pH-sensitive materials swell or collapse depending on the surrounding pH due to the presence of functional groups such as hydroxyl (−OH) group in the polymer chain. Moisture-sensitive materials (e.g., hydrogels) have a similar hydrophilic functional group as the pH-responsive systems. Their hydrophilicity causes the systems to swell to a volume greater than the original volume. 37 38

The ability of 4D-printed prosthetics to adapt to a patient's changing anatomy can significantly enhance comfort and reduce the need for frequent adjustments or replacements. 39 40

The dynamic nature of 4D-printed materials allows for better aesthetic integration with the patient's natural features, improving the prosthetic's visual and functional aspects. Integrating AI and 4D printing in maxillofacial applications can aid in the early detection of medical conditions. This allows for timely intervention and improved patient care. Technology can help identify potential problems before they become more serious. 41

Using 4D printing has enhanced the efficiency of prosthetic production and application. This emerging technology simplifies the manufacturing process by reducing the need for multiple fittings and adjustments. As a result, health care providers can save valuable time and resources. Additionally, this technology allows for faster turnaround times, ultimately enhancing patient satisfaction. 42

The five-dimensional (5D) printing refers to a process where the material is deposited along curved axes (instead of the traditional flat layers in 3DP). This adds more freedom in how the material is arranged, leading to stronger and more complex structure. The material is deposited in five dimensions: *X* , *Y* , *Z* , and two rotational axes ( *A* and *B* ). This results in stronger, lighter facial prosthetic structures with improved integration capabilities. 43

The six-dimensional printing is a conceptual expansion of 5D printing, integrating additional real-time sensory feedback and self-healing capabilities during or after fabrication. It Incorporates six dimensions: *X* , *Y* , *Z* , two rotational axes ( *A* and *B* ), plus functional interactivity (real-time feedback). Smart prosthetics with built-in sensors could be used to monitor fit, functionality, and tissue interaction in maxillofacial prosthetics. 44

### Digital Twin Technology

Digital twin (DT) refers to the creation of a virtual representation of a physical object, system, or process that mirrors its real-world counterpart. This digital replica is connected to the physical entity via sensors and data streams, allowing for real-time monitoring, analysis, and simulation. 45

DT optimizes treatments by simulating their effects on virtual patient models, reducing trial-and-error and adverse outcomes. Surgeons benefit from enhanced surgical planning and training through detailed visualizations of anatomical structures. In drug discovery, DT predicts drug efficacy, safety, and potential side effects based on genetic profiles. Remote patient monitoring becomes feasible as models can be accessed anywhere. Overall, DT significantly enhances patient outcomes, care quality, and operational efficiency. 46 47

Seth et al 48 conducted a systematic review to evaluate the current applications, effectiveness, and limitations of DT technology in plastic surgery. The authors screened 110 studies that specifically focused on DT use in various domains of plastic surgery, including breast reconstruction, craniofacial surgery, and microsurgery. Across these studies, DTs were primarily employed for preoperative planning and intraoperative guidance, with reported benefits including improved surgical accuracy, reduced complications, and enhanced patient satisfaction. Despite promising outcomes, the review identified significant challenges impeding widespread adoption. These include high costs, technical complexity, reliance on advanced imaging and computational technologies, and limited integration in postoperative care or real-time monitoring.

### Virtual Reality and Augmented Reality

The integration of VR and AR into maxillofacial prosthetics offers numerous benefits, from enhanced precision in surgical planning to improved patient engagement and advanced medical training. VR offers a fully immersive digital environment, enabling the replication of surgical and anatomical scenarios for purposes such as education, treatment planning, and patient involvement. In contrast, AR integrates digital information with the physical world, allowing clinicians to visualize 3D models of prostheses and anatomical structures directly within the surgical setting. Together, these technologies provide dynamic tools for real-time simulation and visualization, enhancing surgical precision and improving patient outcomes. 49

In other words, VR transmits the user to a virtual environment, whereas AR overlays digital information on real objects or places in real time. Precise image segmentation is a critical step in digital image processing for VR and AR applications. This process delineates detailed anatomical data and pathological features, such as the extent of tumor infiltration, which is especially important for virtual surgical planning (VSP). 50

These technologies are being explored for patient education and presurgical planning. VR and AR can provide immersive experiences that help patients visualize expected outcomes, leading to better patient engagement and understanding of the treatment process. 51 52

Shepherd et al 53 conducted a scoping review to evaluate the current literature on the use of immersive VR for enhancing patient understanding in perioperative settings. The review focused on studies involving adult patients (≥ 18 years) where VR was employed to support education about their medical condition or upcoming surgical procedure. A total of 12 studies were included, most of which were unpowered and nonrandomized experimental trials. In these studies, VR was primarily utilized during the informed consent process, allowing patients to visualize 3D anatomical models relevant to their surgery. The results consistently indicated improvements in both subjective and objective measures of patient understanding following VR exposure. However, the authors emphasize the need for future research employing rigorous, statistically powered study designs to validate these early findings and support broader clinical integration.

### Virtual Surgical Planning

This technique employs VR and AR technologies to simulate surgical procedures and implant placement. By creating a virtual environment, practitioners can visualize the treatment plan, assess the fit of prosthetic devices, and make necessary adjustments before the actual surgical process as an alternative to conventional navigational systems. 16 54 55

In VSP, VR facilitates the visualization of digital information in three dimensions. The surgeon uses handheld regulators and devices with haptic feedback to interact with the virtual surgical environment and the physical spatial position. VR is extensively used for preoperative anatomical assessment, VSP, and intraoperative navigation. 56

On the other hand, AR enables the operator to overlap digital models and data directly onto the surgical bed. This projection into the real world improves the surgical experience while excluding the need to look away from the patient. 57 AR utilizes several technologies to overlay the physical world viewed by the operator. An optical see-through display uses devices like Google Glass or HoloLens to project the digital-to-real anatomy. The degree of accuracy and precision in genioplasty and orthognathic surgeries planned by HoloLens was acceptable but not satisfactory for mandibular angle osteotomy. 58 59

Integrated planning is another concept that combines surgical planning with prosthodontic rehabilitation, enabling precise prosthesis design. Various CAD/CAM-manufactured tools, such as the surgical guides, support the transition from virtual planning to surgery. 60 Additional tools for dental rehabilitation, such as optical scans of the occlusion or virtual dental setups, may be integrated into the planning process. Superimposing these supplementary elements with virtual 3D models results in highly detailed hybrid models. 50

VSP has proven particularly beneficial for patients with head and neck cancer, improving the accuracy of zygomatic implant positioning and the fit of obturator prostheses. These innovative techniques have also been beneficial in mandibular resection and reconstruction, the restoration of intermaxillary relationships, and dental rehabilitation. 50 52 55

A systematic review by Tel et al, 61 analyzed the software utilized for VSP in craniomaxillofacial surgery over the past decade. The study identified 77 different software programs, with the Materialise suite being the most prevalent, accounting for 36.3% of cases. The review highlighted that certain software packages are associated with specific surgical procedures, underscoring the tailored application of VSP tools in clinical practice.

A systematic review and meta-analysis examined the comparative advantages of VSP over traditional surgical planning. The findings indicated that VSP offers improved accuracy in achieving planned surgical outcomes, particularly in complex bimaxillary osteotomies. However, the evidence regarding reductions in operative time remains inconclusive, suggesting the need for further high-quality studies to substantiate these benefits. 62

Nevertheless, ongoing research is necessary to address existing challenges in VSP, such as standardization of software applications, cost considerations, and the need for comprehensive clinical validation across various surgical disciplines.

### Patient-Specific Implants

Conventional treatments for bone defects, such as standard-sized implants and autogenous bone grafts, are considered the gold standard. However, these methods require customization to fit the shape of the defects, which can be labor-intensive and time-consuming. This challenge is particularly pronounced in the oral and maxillofacial regions, where complex anatomical structures like the orbital floor pose difficulties in obtaining an exact 3D shape. Even minor discrepancies between the implant and the bone defect can lead to implant instability or failure. 33

PSIs that precisely match the original structures in a shorter timeframe have emerged to address these challenges. Notably, 3D-printed titanium implants have gained significant attention, particularly for reconstructing large-sized, load-bearing areas. Titanium implants offer exceptional strength-to-weight ratio, rigidity, biocompatibility, anti-infection, corrosion resistance, and nonmagnetic properties. However, they are often costly and heavier than the original anatomy, leading to subsidence resulting from stress shielding effects and disparities in elasticity modulus. 63 64

Alternatively, researchers have explored using biocompatible polymer materials with reduced weight. Bioresorbable patient-specific 3D-printed bone grafts, meshes, and plates are a promising alternative to conventional implants. 3DP of medical-grade polymer with β-tricalcium phosphate has been used to create degradable polymeric PSIs that were integrated into VSP. It reported desirable surface characteristics and achievable wall thicknesses, enabling the successful fabrication of plates, orbital implants, bone regeneration meshes, and scaffolds. 65 66

Others have focused on optimizing the internal configuration of titanium implants using 3DP methods such as SLM and electron beam melting. These methods allow for designing desired internal configurations and maintaining porosity and pore continuity to reduce implant weight and enhance osteointegration. 67 68 69

These customized implants have demonstrated potential clinical applications in cranioplasty and mandibular reconstruction, providing stability and contributing to the correct implementation of the reconstruction by enabling a completely digital workflow. Compared to conventional reconstruction plates, these PSIs offer further benefits, including high flexibility in plate design and screw placement, reduced operating times, and potential biomechanical improvements, as they may be less prone to plate fatigue fractures. 70

Korn et al 71 evaluated the clinical outcomes of using PSIs for dental rehabilitation in patients who had undergone maxillary resection due to squamous cell carcinoma. All implants demonstrated primary and long-term clinical stability over a mean follow-up period of 26 months. Definitive prosthodontic restorations were completed in every case, with no instances of implant loosening. Major complications such as abscess formation or fistulas were exclusively reported in irradiated patients with poor soft tissue conditions. Minor complications, including mucositis and exposure of the implant framework, were observed but did not affect the functionality or survival of the prostheses.

Short-term results of PSI are promising, but there is a paucity of long-term data and well-conducted randomized clinical trials assessing the durability and overall efficacy of PSIs. Further research is needed to substantiate their long-term benefits and identify potential risks.

### Artificial Intelligence and Machine Learning Algorithms

AI is anticipated to play a significant role in the future of maxillofacial prosthodontics, offering potential enhancements in treatment planning and patient care. AI facilitates automated processing and decision-making, while AR enhances real-world experiences by superimposing digital data, providing new possibilities for patient care. 72 73

Introducing AI with appropriate algorithms to provide suggestions on the crucial parameters such as the most suitable transplant for a specific defect has great potential to automate VSP, significantly reduce the time and effort required, and improve outcomes. 74 75 76

Modabber et al 77 evaluated an algorithm for automating the VSP of fibula transfer, demonstrating that this technology is applicable and can simplify the procedure. Automating digital planning will likely further decrease the costs of VSP. Furthermore, it will simplify the technology, and through this, the surgical procedure of microvascular reconstruction of the mandible may become part of the daily routine of centers and surgeons that do not yet perform it.

Pathak et al 78 employed a combination of AI and traditional methods to fabricate an implant-supported prosthetic ear. The researchers employed AI-generated software to create a symmetrical, natural-looking appearance for the prosthesis. Furthermore, AI techniques facilitated the replication of the human ear's intricate, convoluted, and undulating anatomy. The AI algorithms ensured precise replication through detailed scanning and analysis, restoring symmetry and individuality to the patients' features.

Ali et al 79 evaluated the performance of four pretrained convolutional neural networks in recognizing seven distinct maxillary prosthodontic scenarios, a preliminary step toward developing an AI-powered prosthesis design system. All models consistently achieved diagnostic accuracies exceeding 90%, demonstrating their potential utility in dental image analysis. This AI application could aid in task allocation based on difficulty levels and enable the development of an automated diagnosis system upon patient admission. Furthermore, it facilitates prosthesis design by integrating the necessary morphology, oral function, and treatment complexity.

### Robotics in Maxillofacial Prosthetics

Systems like Yomi, the first Food and Drug Administration-approved robot-assisted dental surgery system, uses real-time navigation and robotic arms to ensure accurate implant placement, minimizing risks like nerve damage and bleeding. Autonomous systems, such as those developed in China, perform minimally invasive surgeries with high precision, reducing recovery times and improving patient safety. These systems integrate features like preoperative planning, real-time navigation, and adaptive force feedback, ensuring accurate and efficient procedures. Additionally, collaborative human-robot systems combine the surgeon's expertise with robotic stability, enabling safer and more flexible operations even in complex cases. 80 81 82

Although direct studies on robotics in maxillofacial prosthetics are scarce, robotic systems have been utilized in related maxillofacial procedures. Jain et al 83 assessed the accuracy of robot-assisted implant placement compared to dynamic and static computer-assisted implant surgeries. The findings indicated that robotic systems achieved significantly lower angular deviations and slightly reduced coronal and apical deviations, suggesting enhanced precision over traditional methods.

A multicenter randomized controlled trial evaluated a semiactive robotic system for dental implants. Results demonstrated superior accuracy compared to freehand surgery. 84

While robotics have demonstrated enhanced precision in dental implantology and certain maxillofacial surgeries, their direct application in maxillofacial prosthetics is still emerging. Further research is needed to evaluate the effectiveness and practicality of integrating robotic systems into the design and fabrication of maxillofacial prostheses.

### Teleprosthetics and Remote Consultations

These technologies facilitate remote consultations and virtual simulations, improving accessibility and patient engagement in treatment planning. 85

The digital approach allowing clinicians to refine treatment plans before physical fabrication, optimize outcomes and minimize the need for adjustments. 13 15

The coronavirus disease 2019 pandemic has accelerated the adoption of telemedicine in maxillofacial rehabilitation. Remote consultations allow for ongoing patient management and follow-up, thereby improving access to care for patients in remote locations. 86

Despite the potential benefits, there is a notable scarcity of researches specifically addressing teleprosthetics in maxillofacial prosthetics. Mariño and Ghanim 87 highlighted that teledentistry applications, such as remote consultations and diagnoses, have been successfully implemented across multiple countries, primarily focusing on education and diagnostic services. These findings suggest a foundational framework that could be adapted for maxillofacial prosthetics.

Moosa et al 87 conducted a cross-sectional study to evaluate the perceptions and attitudes of dental professionals in Pakistan toward teledentistry, focusing on four domains: data security and consent, practice enhancement, usefulness in clinical settings, and patient benefits. The study revealed notable concerns regarding data security (59.8%) and patient consent (52%), yet a majority (61.8%) acknowledged teledentistry's potential to reduce waiting times and enhance clinical efficiency.

Further research is necessary to assess the feasibility, efficacy, and patient outcomes of teleprosthetics interventions in maxillofacial care.

## Future Research Directions

Despite notable advancements in maxillofacial prosthetics, several areas require further investigation to support evidence-based clinical practices. Long-term clinical studies are essential to evaluate the durability, functional stability, and patient satisfaction of various prosthetic materials and patient-specific designs used in maxillofacial rehabilitation. Standardized treatment protocols based on robust data are still lacking. Additionally, integrating emerging technologies such as robotics, AI, and mixed reality remains limited and calls for exploration to enhance precision in surgical planning, prosthesis fabrication, and intraoperative navigation.

Another significant gap lies in the application of telemedicine in maxillofacial prosthetic care. While digital workflows and virtual planning are increasingly adopted, the use of teleprosthetics and remote consultations remains underdeveloped. Future research should evaluate their feasibility, especially for follow-up care and remote patient management.

## Limitations

A primary limitation of this review lies in the heterogeneity of methodologies and reporting standards employed across the included studies. The diverse approaches to assessing and quantifying accuracy in innovative and digital maxillofacial prosthetics, encompassing variations in measurement techniques, sample sizes, and outcome metrics, impede a direct quantitative comparison between digital and conventional fabrication methods. Future research should prioritize developing and adopting standardized protocols for accurate assessment, facilitating more robust meta-analyses and evidence-based comparisons. Furthermore, more clinical trials that directly compare digital and conventional methods, using validated outcome measures, are needed to assess both accuracy and patient-centered outcomes.

## Conclusion

Advanced digital technologies directly affect maxillofacial prosthetic rehabilitation, offering clinicians and patients a new era of more precise, predictable, and efficient treatment options. Digital technologies have streamlined the workflow, from initial imaging and planning and VSP to the final fabrication and customization of the prosthetic device. Integrating these advanced techniques has improved aesthetic and functional outcomes and enhanced patient comfort and compliance throughout rehabilitation.

Recent materials and technology enabled PSIs and bioresorbable patient-specific 3D-printed bone grafts, meshes, and plates. AI algorithms can aid in decision-making and treatment planning. The full potential of digital innovations in maxillofacial prosthetics is still being explored and ongoing research and development of innovative tools and materials are required for further improvements in maxillofacial prosthetics and to enhance the quality of life for individuals in need of these essential restorations.

## References

[1] The Glossary of Prosthodontic Terms 2023: Tenth Edition J Prosthet Dent 2023 130(4, Suppl 1):e7 e126 37914442 10.1016/j.prosdent.2023.03.002

[2] de Caxias F P Dos Santos D M Bannwart L C de Moraes Melo Neto C L Goiato M C Classification, history, and future prospects of maxillofacial prosthesis Int J Dent 2019 2019 8.657619E6 10.1155/2019/8657619 PMC6668529 31396279

[3] Rokohl A C Pine K R Pine N S Prosthetic eye care - the current state of the art Prog Retin Eye Res 2025 105 101337 39938676 10.1016/j.preteyeres.2025.101337

[4] Choubisa D A comprehensive review of extraoral maxillofacial material: part II: early extraoral maxillofacial materials J Dental Res Rev 2023 9 267 275

[5] Singi S R Sathe S Reche A R Sibal A Mantri N Extended arm of precision in prosthodontics: artificial intelligence Cureus 2022 14 11 e30962 36465202 10.7759/cureus.30962 PMC9711888

[6] Luo X Niu J Su G Research progress of biomimetic materials in oral medicine J Biol Eng 2023 17 01 72 37996886 10.1186/s13036-023-00382-4 PMC10668381

[7] Alsulimani O Alhaddad A Altassan M Bukhari A Munshi L Sabir G The precision of all-on-four implant position recorded from three different CBCT machines Eur J Dent 2025 19 02 337 345 39043211 10.1055/s-0044-1788613 PMC12020579

[8] Jeong M Radomski K Lopez D Liu J T Lee J D Lee S J Materials and applications of 3D printing technology in dentistry: an overview Dent J (Basel) 2023 12 01 1 38275676 10.3390/dj12010001 PMC10814684

[9] Isakov T M Härkönen H Atkova I From challenges to opportunities: digital transformation in hospital-at-home care Int J Med Inform 2024 192 105644 39393125 10.1016/j.ijmedinf.2024.105644

[10] Scaringi R Nannelli M Franchina A Full zirconia implant-born prosthetic rehabilitation with CAD/CAM technology after accurate digital planning. A case report Int J Environ Res Public Health 2021 18 15 7998 34360288 10.3390/ijerph18157998 PMC8345593

[11] Wang J An Y X Shi Y L A digital workflow to predict facial aesthetics in patients with maxillofacial trauma with implant retained prostheses J Prosthodont Res 2023 67 03 481 486 36682789 10.2186/jpr.JPR_D_22_00112

[12] Suresh N Janakiram C Nayar S Krishnapriya V N Mathew A Effectiveness of digital data acquisition technologies in the fabrication of maxillofacial prostheses - a systematic review J Oral Biol Craniofac Res 2022 12 01 208 215 35024329 10.1016/j.jobcr.2021.12.004 PMC8733177

[13] Edher F Innovations in fixed prosthodontic workflows J Prosthet Dent 2022 128 04 545 547 36309467 10.1016/j.prosdent.2022.09.001

[14] Budati M Maiti S Digital Workflow in Maxillofacial Prosthetics. Futuristic Trends in Medical Sciences. Vol. 3 New Delhi, India Iterative International Publisher 2023,pp.196 201

[15] Vosselman N Glas H H Merema B J Three-dimensional guided zygomatic implant placement after maxillectomy J Pers Med 2022 12 04 588 35455704 10.3390/jpm12040588 PMC9027393

[16] Bhujbal R B Bakrolwala H Jurel S K Chand P Singh R D Digitalization in maxillofacial prosthodontics- a review The Journal of Dental Panacea. 2023 5 02 59 64

[17] Corsalini M Barile G Ranieri F Comparison between conventional and digital workflow in implant prosthetic rehabilitation: a randomized controlled trial J Funct Biomater 2024 15 06 149 149 38921523 10.3390/jfb15060149 PMC11204927

[18] Aponte-Wesson R Khadivi A A Cardoso R Chambers M S An alternative impression technique for capturing anatomic undercuts to rehabilitate a patient with a total maxillectomy: a clinical report J Prosthet Dent 2019 122 04 412 416 30982617 10.1016/j.prosdent.2019.03.001

[19] Unkovskiy A Spintzyk S Kiemle T Roehler A Huettig F Trueness and precision of skin surface reproduction in digital workflows for facial prosthesis fabrication J Prosthet Dent 2023 130 03 402 413 35256182 10.1016/j.prosdent.2021.06.050

[20] Stuani V T Ferreira R Manfredi G GP Cardoso M V Sant'Ana A CP Photogrammetry as an alternative for acquiring digital dental models: a proof of concept Med Hypotheses 2019 128 43 49 31203907 10.1016/j.mehy.2019.05.015

[21] Baghani M T Neshati A Sadafi M Shidfar S Evaluation of the accuracy of digital and conventional implant-level impression techniques for maxillofacial prosthesis J Family Med Prim Care 2023 12 03 446 451 37122657 10.4103/jfmpc.jfmpc_1324_22 PMC10131967

[22] Hatamleh M M Hatamlah H M Nuseir A Maxillofacial prosthetics and digital technologies: cross-sectional study of healthcare service provision, patient attitudes, and opinions J Prosthodont 2024 33 03 231 238 37218377 10.1111/jopr.13718

[23] Ahmad S Hasan N Review on 3D printing in dentistry: conventional to personalized dental care J Biomater Sci Polym Ed 2022 33 17 2292 2323 35796720 10.1080/09205063.2022.2099666

[24] Tian Y Chen C Xu X A review of 3D printing in dentistry: technologies, affecting factors, and applications Scanning 2021 2021 9.950131E6 10.1155/2021/9950131 PMC8313360 34367410

[25] Zandinejad A Yilmaz B 3D printing in clinical dentistry Int J Prosthodont 2024 37 07 3 38489215 10.11607/ijp.2024.s1.e

[26] Geusens J Sun Y Luebbers H T Bila M Darche V Politis C Accuracy of computer-aided design/computer-aided manufacturing-assisted mandibular reconstruction with a fibula free flap J Craniofac Surg 2019 30 08 2319 2323 31261320 10.1097/SCS.0000000000005704

[27] Zeller A N Neuhaus M T Weissbach L VM Patient-specific mandibular reconstruction plates increase accuracy and long-term stability in immediate alloplastic reconstruction of segmental mandibular defects J Maxillofac Oral Surg 2020 19 04 609 615 33071511 10.1007/s12663-019-01323-9 PMC7524954

[28] Costa-Palau S Clua-Palau A Real-Voltas F Brufau-de Barbera M Cabratosa-Termes J A comparison of digital and conventional fabrication techniques for an esthetic maxillofacial prosthesis for the cheek and lip J Prosthet Dent 2025 133 01 315 320 36872157 10.1016/j.prosdent.2023.01.020

[29] Ali I E Enomoto K Sumita Y Wakabayashi N Combined digital-conventional workflow to fabricate a definitive obturator from an interim obturator for a patient with an anterior maxillectomy defect J Prosthet Dent 2025 133 03 920 925 37277237 10.1016/j.prosdent.2023.04.028

[30] Zoabi A Redenski I Oren D 3D printing and virtual surgical planning in oral and maxillofacial surgery J Clin Med 2022 11 09 2385 35566511 10.3390/jcm11092385 PMC9104292

[31] Bansod A V Pisulkar S G Dahihandekar C Beri A Rapid prototyping in maxillofacial rehabilitation: a review of literature Cureus 2022 14 09 e28969 36237787 10.7759/cureus.28969 PMC9548214

[32] Kongsong W Sittitavornwong S Utilization of virtual surgical planning for surgical splint-assisted comminuted maxillomandibular fracture reduction and/or fixation Craniomaxillofac Trauma Reconstr 2020 13 04 334 341 33456705 10.1177/1943387520948677 PMC7797978

[33] Wang X Mu M Yan J Han B Ye R Guo G 3D printing materials and 3D printed surgical devices in oral and maxillofacial surgery: design, workflow and effectiveness Regen Biomater 2024 11 rbae066 39169972 10.1093/rb/rbae066 PMC11338467

[34] Kouhi M de Souza Araújo I J Asa'ad F Recent advances in additive manufacturing of patient-specific devices for dental and maxillofacial rehabilitation Dent Mater 2024 40 04 700 715 38401992 10.1016/j.dental.2024.02.006 PMC13011888

[35] Afzali Naniz M Askari M Zolfagharian A Afzali Naniz M Bodaghi M 4D printing: a cutting-edge platform for biomedical applications Biomed Mater 2022 17 06 62001 62001 10.1088/1748-605X/ac8e42 36044881

[36] Gharehdaghi N Nokhbatolfoghahaei H Khojasteh A 4D printing of smart scaffolds for bone regeneration: a systematic review Biomed Mater 2024 20 01 12003 10.1088/1748-605X/ad8f80 39504649

[37] Pourmasoumi P Moghaddam A Nemati Mahand S A review on the recent progress, opportunities, and challenges of 4D printing and bioprinting in regenerative medicine J Biomater Sci Polym Ed 2023 34 01 108 146 35924585 10.1080/09205063.2022.2110480

[38] Lai J Liu Y Lu G 4D bioprinting of programmed dynamic tissues Bioact Mater 2024 37 348 377 38694766 10.1016/j.bioactmat.2024.03.033 PMC11061618

[39] Palanisamy S Exploring the horizons of four-dimensional printing technology in dentistry Cureus 2024 16 04 e58572 38770499 10.7759/cureus.58572 PMC11102886

[40] Perambudhuru Y Goyal L Dewan M Mahajan A Chaudhari P K Application of 4D printing in dentistry: a narrative review J Adv Periodontol Implant Dent 2024 16 01 55 63 39027206 10.34172/japid.2024.003 PMC11252150

[41] Grillo R Reis B AQ Lima B C Melhem-Elias F Shaping the 4D frontier in maxillofacial surgery with faceMesh evolution J Stomatol Oral Maxillofac Surg 2024 125(3S):101843 101843 38521241 10.1016/j.jormas.2024.101843

[42] Revathi S Babu M Rajkumar N Vinod Kumar V M Sumanth Ratna K Sampath B Unleashing the Future Potential of 4D Printing: Exploring Applications in Wearable Technology, Robotics, Energy, Transportation, and Fashion Hershey, PA IGI Global 2024 131 153

[43] Haleem A Javaid M Vaishya R 5D printing and its expected applications in orthopaedics J Clin Orthop Trauma 2019 10 04 809 810 31316262 10.1016/j.jcot.2018.11.014 PMC6611828

[44] Vasiliadis A V Koukoulias N Katakalos K From three-dimensional (3D)- to 6D-printing technology in orthopedics: science fiction or scientific reality? J Funct Biomater 2022 13 03 101 35893469 10.3390/jfb13030101 PMC9326671

[45] Klimo M Kvassay M Kvaššayová N Digital Twin and Modelling a 3D Human Body in Healthcare21st International Conference on Emerging e Learning Technologies and Applications (ICETA). 2023. pp. 307-312

[46] Martinez-Velazquez R Gamez R El Saddik A Cardio Twin: A Digital Twin of the human heart running on the edgeIEEE International Symposium on Medical Measurements and Applications (MeMeA). Istanbul, Turkey, 2019, pp. 1-6

[47] Braun M Represent me: please! Towards an ethics of digital twins in medicineJ Med Ethics2021:medethics-2020-106134 10.1136/medethics-2020-106134 33722986

[48] Seth I Lim B Lu P YJ Digital Twins use in plastic surgery: a systematic review J Clin Med 2024 13 24 7861 39768784 10.3390/jcm13247861 PMC11728120

[49] Ayoub A Pulijala Y The application of virtual reality and augmented reality in oral & maxillofacial surgery BMC Oral Health 2019 19 01 238 31703708 10.1186/s12903-019-0937-8 PMC6839223

[50] Probst F A Liokatis P Mast G Ehrenfeld M Virtual planning for mandible resection and reconstruction Innov Surg Sci 2023 8 03 137 148 38077486 10.1515/iss-2021-0045 PMC10709695

[51] Khan H U Ali Y Khan F Al-Antari M A A comprehensive study on unraveling the advances of immersive technologies (VR/AR/MR/XR) in the healthcare sector during the COVID-19: challenges and solutions Heliyon 2024 10 15 e35037 39157361 10.1016/j.heliyon.2024.e35037 PMC11328097

[52] Iqbal A I Aamir A Hammad A Immersive technologies in healthcare: an in-depth exploration of virtual reality and augmented reality in enhancing patient care, medical education, and training paradigmsJ Prim Care Community Health2024 15:21501319241293311 10.1177/21501319241293311 PMC11528804 39439304

[53] Shepherd T Trinder M Theophilus M Does virtual reality in the perioperative setting for patient education improve understanding? A scoping review Surg Pract Sci 2022 10 100101 39845596 10.1016/j.sipas.2022.100101 PMC11749402

[54] Elbashti M E Itamiya T Aswehlee A M Sumita Y I Ella B Naveau A Augmented reality for interactive visualization of 3D maxillofacial prosthetic data Int J Prosthodont 2020 33 06 680 683 33284911 10.11607/ijp.6835

[55] Stucki J Dastgir R Baur D A Quereshy F A The use of virtual reality and augmented reality in oral and maxillofacial surgery: a narrative review Oral Surg Oral Med Oral Pathol Oral Radiol 2024 137 01 12 18 37723007 10.1016/j.oooo.2023.07.001

[56] Bartella A K Kamal M Scholl I Virtual reality in preoperative imaging in maxillofacial surgery: implementation of “the next level”? Br J Oral Maxillofac Surg 2019 57 07 644 648 31204187 10.1016/j.bjoms.2019.02.014

[57] De Paolis L T De Luca V Augmented visualization with depth perception cues to improve the surgeon's performance in minimally invasive surgery Med Biol Eng Comput 2019 57 05 995 1013 30511205 10.1007/s11517-018-1929-6

[58] Fushima K Kobayashi M Mixed-reality simulation for orthognathic surgery Maxillofac Plast Reconstr Surg 2016 38 01 13 27014664 10.1186/s40902-016-0059-z PMC4783436

[59] Brunzini A Mandolini M Caragiuli M Germani M Mazzoli A Pagnoni M HoloLens 2 for maxillofacial surgery: a preliminary study Cham: Springer 2022pp123 134

[60] Vosselman N Alberga J Witjes M HJ Prosthodontic rehabilitation of head and neck cancer patients-challenges and new developments Oral Dis 2021 27 01 64 72 32343862 10.1111/odi.13374 PMC7818410

[61] Tel A Arboit L De Martino M Isola M Sembronio S Robiony M Systematic review of the software used for virtual surgical planning in craniomaxillofacial surgery over the last decade Int J Oral Maxillofac Implants 2023 52 07 775 786 10.1016/j.ijom.2022.11.011 36481124

[62] Alkaabi S Maningky M Helder M N Alsabri G Virtual and traditional surgical planning in orthognathic surgery - systematic review and meta-analysis Br J Oral Maxillofac Surg 2022 60 09 1184 1191 36030091 10.1016/j.bjoms.2022.07.007

[63] Goodson A MC Parmar S Ganesh S Printed titanium implants in UK craniomaxillofacial surgery. Part II: perceived performance (outcomes, logistics, and costs) Br J Oral Maxillofac Surg 2021 59 03 320 328 33280945 10.1016/j.bjoms.2020.08.088

[64] Goodson A MC Parmar S Ganesh S Printed titanium implants in UK craniomaxillofacial surgery. Part I: access to digital planning and perceived scope for use in common procedures Br J Oral Maxillofac Surg 2021 59 03 312 319 33280946 10.1016/j.bjoms.2020.08.087

[65] Shilo D Capucha T Goldstein D Bereznyak Y Emodi O Rachmiel A Treatment of facial deformities using 3D planning and printing of patient-specific implants J Vis Exp 2020 159 e60930 10.3791/60930 32510490

[66] Maintz M Tourbier C de Wild M Patient-specific implants made of 3D printed bioresorbable polymers at the point-of-care: material, technology, and scope of surgical application 3D Print Med 2024 10 01 13 38639834 10.1186/s41205-024-00207-0 PMC11031859

[67] Sharma N Ostas D Rotar H Brantner P Thieringer F M Design and additive manufacturing of a biomimetic customized cranial implant based on Voronoi diagram Front Physiol 2021 12 647923 33897455 10.3389/fphys.2021.647923 PMC8063040

[68] Lee U L Yun S Lee H Osseointegration of 3D-printed titanium implants with surface and structure modifications Dent Mater 2022 38 10 1648 1660 36075761 10.1016/j.dental.2022.08.003

[69] Sadowsky S J Has zirconia made a material difference in implant prosthodontics? A review Dent Mater 2020 36 01 1 8 31500904 10.1016/j.dental.2019.08.100

[70] Major R Kowalczyk P Surmiak M Patient specific implants for jawbone reconstruction after tumor resection Colloids Surf B Biointerfaces 2020 193 111056 32403035 10.1016/j.colsurfb.2020.111056

[71] Korn P Gellrich N C Jehn P Spalthoff S Rahlf B A new strategy for patient-specific implant-borne dental rehabilitation in patients with extended maxillary defects Front Oncol 2021 11 718872 34956858 10.3389/fonc.2021.718872 PMC8708135

[72] Zatt F P Rocha A O Anjos L MD Caldas R A Cardoso M Rabelo G D Artificial intelligence applications in dentistry: a bibliometric review with an emphasis on computational research trends within the field J Am Dent Assoc 2024 155 09 755 7.64E7 39093229 10.1016/j.adaj.2024.05.013

[73] Rahim A Khatoon R Khan T A Artificial intelligence-powered dentistry: probing the potential, challenges, and ethicality of artificial intelligence in dentistryDigit Health2024 10:20552076241291345 10.1177/20552076241291345 PMC11558748 39539720

[74] Miragall M F Knoedler S Kauke-Navarro M Face the future-artificial intelligence in oral and maxillofacial surgery J Clin Med 2023 12 21 6843 37959310 10.3390/jcm12216843 PMC10649053

[75] Wolfaardt J F Brecht L E Taft R M The future of maxillofacial prosthodontics in North America: part II - a survey J Prosthet Dent 2022 127 02 351 357 33431174 10.1016/j.prosdent.2020.11.013

[76] Wolfaardt J F Brecht L E Taft R M The future of maxillofacial prosthodontics in North America: part I-journey to the present J Prosthet Dent 2022 127 02 345 350 33431175 10.1016/j.prosdent.2020.11.012

[77] Modabber A Rauen A Ayoub N Evaluation of a novel algorithm for automating virtual surgical planning in mandibular reconstruction using fibula flaps J Craniomaxillofac Surg 2019 47 09 1378 1386 31331845 10.1016/j.jcms.2019.06.013

[78] Pathak A Dhamande M M Gujjelwar S Das P Chheda E V Puthenkandathil R Fabrication of implant-supported auricular prosthesis using artificial intelligence Cureus 2024 16 05 e60267 38872639 10.7759/cureus.60267 PMC11170235

[79] Ali I E Sumita Y Wakabayashi N Advancing maxillofacial prosthodontics by using pre-trained convolutional neural networks: image-based classification of the maxilla J Prosthodont 2024 33 07 645 654 38566564 10.1111/jopr.13853

[80] Ke Y F Zhang Y P Wang Y Sun Y C Application and outlook of robotics in prosthetic dentistry [in Chinese] Zhonghua Kou Qiang Yi Xue Za Zhi 2021 56 09 939 944 34496546 10.3760/cma.j.cn112144-20200924-00512

[81] Grischke J Johannsmeier L Eich L Griga L Haddadin S Dentronics: towards robotics and artificial intelligence in dentistry Dent Mater 2020 36 06 765 778 32349877 10.1016/j.dental.2020.03.021

[82] Liu L Watanabe M Ichikawa T Robotics in dentistry: a narrative review Dent J 2023 11 03 62 10.3390/dj11030062 PMC10047128 36975559

[83] Jain S Sayed M E Ibraheem W I Accuracy comparison between robot-assisted dental implant placement and static/dynamic computer-assisted implant surgery: a systematic review and meta-analysis of in vitro studies Medicina (Kaunas) 2023 60 01 11 38276045 10.3390/medicina60010011 PMC10817552

[84] Yang F Chen J Cao R Comparative analysis of dental implant placement accuracy: Semi-active robotic versus free-hand techniques: a randomized controlled clinical trial Clin Implant Dent Relat Res 2024 26 06 1149 1161 39161058 10.1111/cid.13375 PMC11660539

[85] Prajapati D P Maxillofacial Prosthodontics: Present and Future. Futuristic Trends in Medical SciencesVol. 3.New Delhi, India Iterative International Publisher 2024.pp.200 206

[86] Hung M Lipsky M S Phuatrakoon T N Nguyen M Licari F W Unni E J Teledentistry implementation during the COVID-19 pandemic: scoping review Interact J Med Res 2022 11 02 e39955 35862174 10.2196/39955 PMC9307266

[87] Mariño R Ghanim A Teledentistry: a systematic review of the literature J Telemed Telecare 2013 19 04 179 183 23512650 10.1177/1357633x13479704

[88] Moosa Y Samaranayake L P Pisarnturakit P P Perception and attitudes of dental professionals on teledentistry: a cross-sectional study Eur J Dent 2025 19 04 1134 1145 40073989 10.1055/s-0044-1801301 PMC12494421

